# Antagonistic analogs of growth hormone-releasing hormone increase the efficacy of treatment of triple negative breast cancer in nude mice with doxorubicin; A preclinical study

**DOI:** 10.18632/oncoscience.92

**Published:** 2014-10-24

**Authors:** Roberto Perez, Andrew V Schally, Petra Popovics, Renzhi Cai, Wei Sha, Ricardo Rincon, Ferenc G. Rick

**Affiliations:** ^1^ Veterans Affairs Medical Center, Miami, FL; ^2^ South Florida VA Foundation for Research and Education, Miami, FL; ^3^ Department of Pathology, University of Miami, Miller School of Medicine, Miami, FL; ^4^ Division of Hematology/Oncology, University of Miami, Miller School of Medicine, Miami, FL; ^5^ Division of Endocrinology, Department of Medicine, University of Miami, Miller School of Medicine, Miami, FL; ^6^ Sylvester Comprehensive Cancer Center, Miller School of Medicine, University of Miami, Miami, FL; ^7^ Division of Cardiology, Department of Medicine, Miller School of Medicine, University of Miami, Miami, FL; ^8^ Department of Medicine III, Technical University Dresden, Dresden, Germany; ^9^ Department of Urology, Herbert Wertheim College of Medicine, Florida International University, Miami, FL

**Keywords:** triple negative breast cancer, drug resistance, combination therapy, growth-hormone-releasing hormone, antagonist, GHRH analogs

## Abstract

**Introduction:**

This study evaluated the effects of an antagonistic analog of growth hormone-releasing hormone, MIA-602, on tumor growth, response to doxorubicin, expression of drug resistance genes, and efflux pump function in human triple negative breast cancers.

**Methods:**

HCC1806 (doxorubicin-sensitive) and MX-1 (doxorubicin-resistant), cell lines were xenografted into nude mice and treated with MIA-602, doxorubicin, or their combination. Tumors were evaluated for changes in volume and the expression of the drug resistance genes MDR1 and NANOG. *In-vitro* cell culture assays were used to analyze the effect of MIA-602 on efflux pump function.

**Results:**

Therapy with MIA-602 significantly reduced tumor growth and enhanced the efficacy of doxorubicin in both cell lines. Control HCC1806 tumors grew by 435%, while the volume of tumors treated with MIA-602 enlarged by 172.2% and with doxorubicin by 201.6%. Treatment with the combination of MIA-602 and doxorubicin resulted in an increase in volume of only 76.2%. Control MX-1 tumors grew by 907%, while tumors treated with MIA-602 enlarged by 434.8% and with doxorubicin by 815%. The combination of MIA-602 and doxorubicin reduced the increase in tumor volume to 256%. Treatment with MIA-602 lowered the level of growth hormone-releasing hormone and growth hormone-releasing hormone receptors and significantly reduced the expression of multidrug resistance (MDR1) gene and the drug resistance regulator NANOG. MIA-602 also suppressed efflux pump function in both cell lines.

**Conclusions:**

We conclude that treatment of triple negative breast cancers with growth hormone-releasing hormone antagonists reduces tumor growth and potentiates the effects of cytotoxic therapy by nullifying drug resistance.

## INTRODUCTION

In the United States alone, nearly 200,000 women are afflicted with breast cancer each year and 41,000 die as a result of their malignancy.[1] Breast cancer is the leading cause of mortality in Hispanic and African-American women and the second most common cause of cancer-related death of women. The US figures can be extrapolated to approximately 4 million new cases and 820,000 deaths per year, worldwide.

Breast cancer is a very heterogeneous disease. The subtype defined as triple negative breast cancer (TNBC) is negative for estrogen receptor, progesterone receptor, and the human epidermal growth factor receptor 2 (Her2).[2] TNBC accounts for 10-15% of all breast cancer cases and has a higher rate of mortality than other malignancies. The TNBC phenotype is hereditary, affects younger women, is more invasive, and has a much poorer prognosis.[3] These cancers are extremely resistant to the treatment options available for other breast cancers, with drug efflux being the primary mechanism of resistance.[4] This accounts for the low survival rate of women with TNBC.[5] Alternate treatment strategies must therefore be devised to address this clinical deficiency.

Growth hormone releasing hormone (GHRH) is a neuropeptide hormone, secreted by the hypothalamus, which regulates the synthesis and release of growth hormone by the pituitary.[6] Growth hormone subsequently stimulates the release of hepatic insulin-like growth factor, which is a major anabolic growth factor and a potent mitogen for many neoplasms.[7-9] Additionally, GHRH and GHRH receptors (GHRH-R) are not confined to the hypothalamic–pituitary axis, but are also produced by various extra-hypothalamic sites.[10-16] Biologically active GHRH, mRNA for GHRH, GHRH-R, and splice variants of GHRH-R have been identified in surgical specimens and tumor cell lines of a multitude of human cancers, including various types of breast cancer. [17-28] Much evidence indicates that GHRH acts as an autocrine/paracrine growth factor in many human cancers [6, 23, 29-31] including that of the breast.[32] Pituitary-type GHRH-R and splice variant 1 of GHRH-R appear to mediate the direct effects of GHRH and its analogs on tumors.[33] *In vitro* and *in vivo* proliferation of various human cancers is suppressed by antagonistic analogs of GHRH (referred to as “GHRH antagonists”).[8, 34, 35] These findings support the concept of GHRH as a growth factor for tumors and suggest that GHRH-R could be used as a suitable therapeutic target.

We have recently reported that the GHRH antagonist, MIA-602, suppresses the expression of inflammatory cytokines in human TNBC tumors xenografted into nude mice.[36] Cytokines have been shown to play a major role in the cellular signaling involved in the pathogenesis of breast cancer.[37-39] In this study, we used HCC1806 and doxorubicin-resistant MX-1 human TNBC cell lines xenografted into nude mice to evaluate the effects of a GHRH antagonist (MIA-602) alone and in combination with doxorubicin. We examined the effects of treatment on tumor growth, drug resistance, GHRH-R levels, expression of MDR1 and Nanog, and efflux pump activity.

## RESULTS

### Effect of treatment with MIA-602, doxorubicin, or their combination on the growth of xenografts of HCC1806 and MX-1 human TNBC

**Figure 1:**
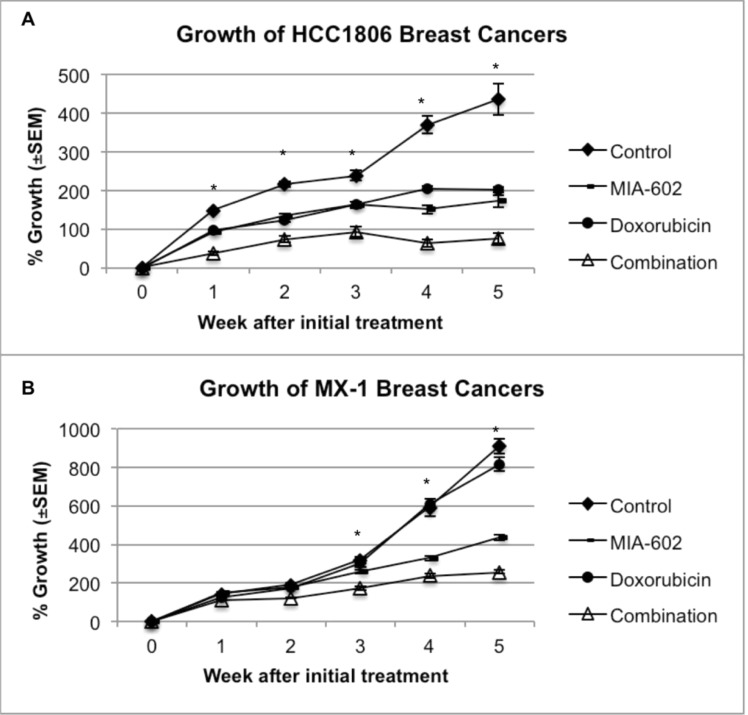
**A.** HCC1806 tumors responded significantly better to combination treatment than to either MIA-602 or doxorubicin alone. **B**. Treatment of MX-1 tumors (doxorubicin resistant) with doxorubicin/MIA-602 combination resulted in elimination of resistance to doxorubicin. Vertical bars indicate ± SEM. * Combination vs. control (P < 0.001), MIA-602 (P < 0.05), and doxorubicin (P < 0.001). N= 16 tumors.

Treatment of nude mice bearing human TNBC tumors was initiated after the tumors had reached a volume of ~100 mm^3^ and lasted for five weeks. Control HCC1806 tumors grew by 435.3% (±41.6%) of the initial tumor volume by week five, while tumors treated with MIA-602 augmented by only 172.2% (±15.1%), significantly (P < 0.001) less than controls. Tumor volume of mice given doxorubicin increased by 201.6% (±7.1%) and was also significantly (P < 0.001) reduced compared to controls. Thus, treatment with MIA-602 reduced the growth of HCC1806 tumors by 60% and doxorubicin by 54% compared to controls. The combination of MIA-602 and doxorubicin had the greatest inhibitory effect and diminished the growth of HCC1806 tumors by 83% versus controls. The treatment with the combination of MIA-602 and doxorubicin resulted in the smallest increase in tumor volume of 76.2% (±13.6%). The growth of tumors treated with the combination was significantly smaller than that of controls (P < 0.001) and tumors treated with either MIA-602 (P < 0.05) or doxorubicin (P < 0.001) (figure 1a).

Control MX-1 tumors grew by 907.4% (±37.4%) of the initial tumor volume by week five, but those treated with MIA-602 increased by 434.8% (±12.9%); significantly (P < 0.001) less than controls. Not surprisingly, the growth of tumors treated with doxorubicin, which attained a volume 814.9% (±34.7%) of the initial size, was not significantly different than controls. Treatment with the combination of MIA-602 and doxorubicin led to an increase in final tumor volume of only 256.0% (±10.3%). Treatment with MIA-602 reduced the growth of MX-1 tumors by 52% and doxorubicin by only 10% compared to controls. The combination of MIA-602 and doxorubicin decreased growth of MX-1 tumors by 72%. Combination therapy resulted in significantly reduced growth compared to controls (P < 0.001), and tumors treated with MIA-602 (P < 0.05) or doxorubicin (P < 0.001) (figure 1b).

### Expression of GHRH and GHRH-R genes by xenografts of HCC1806 and MX-1 human TNBC

**Figure 2:**
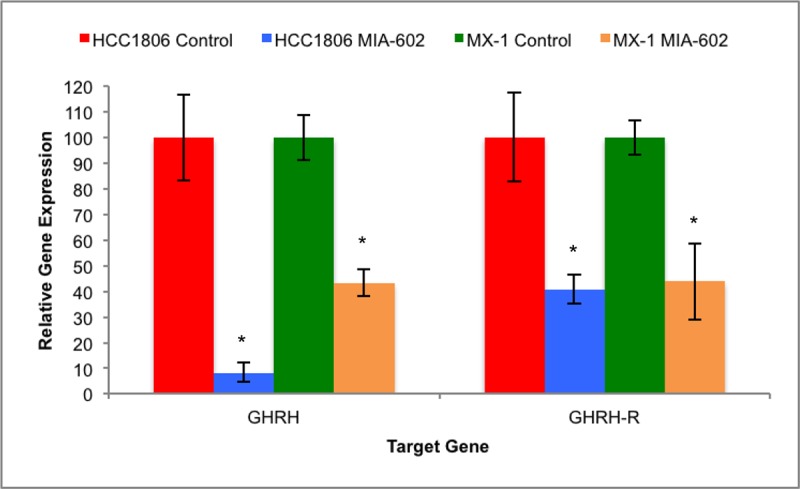
Treatment of HCC1806 and MX-1 human TNBC tumors with the GHRH antagonist MIA-602 significantly suppressed the expression of GHRH and GHRH-R genes Vertical bars indicate ± SEM, n=12 tumors, * *P* < 0.01 vs. control.

Protein and mRNA for GHRH and GHRH-R were expressed in both HCC1806 and MX-1 human TNBC cell lines as determined after five weeks of treatment using qRT-PCR. The expression of GHRH and GHRH-R genes by HCC1806 tumors was significantly (*P* < 0.05) suppressed by treatment, *in-vivo*, with MIA-602. HCC1806 tumors treated with the GHRH antagonist for five weeks expressed 91.8% (±3.8%) less GHRH and the levels of GHRH-R were 59.4% (±5.7%) lower than in controls. Expression of GHRH and GHRH-R genes by MX-1 tumors was also significantly (*P* < 0.05) reduced by therapy with MIA-602. MX-1 tumors treated with the GHRH antagonist for five weeks expressed 56.2% (±5.2%) less GHRH and 56.1% (±14.9%) less GHRH-R than controls (figure 2).

### Expression of regulatory genes of drug resistance by xenografts of HCC1806 and MX-1 human TNBC

The expression of the tumoral multidrug resistance gene (MDR1), which encodes P-glycoprotein, and NANOG, a stem-cell marker and a regulator of the expression of drug resistance genes, was determined using qRT-PCR.

**Figure 3:**
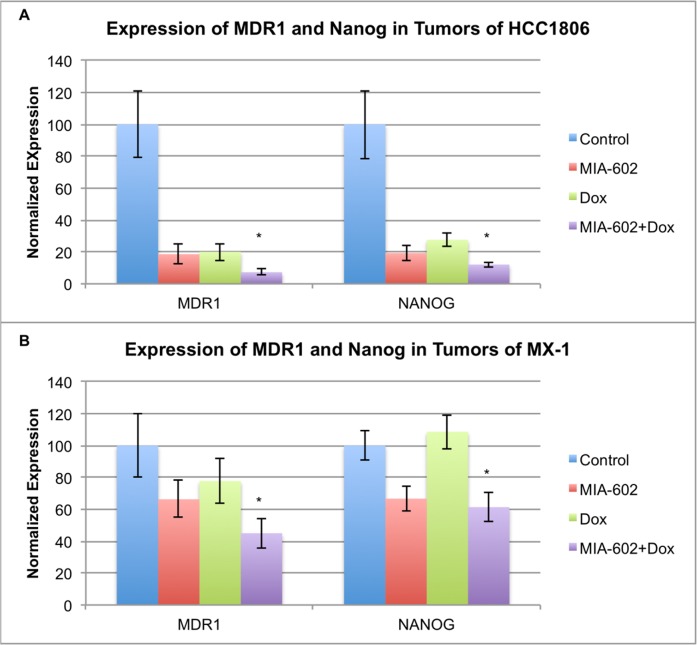
Expression of both NANOG and MDR1 was significantly suppressed by treatment with the MIA-602/doxorubicin combination compared to MIA-602 or doxorubicin alone in A HCC1806 and doxorubicin alone in **B**. doxorubicin resistant MX-1. Vertical bars indicate ± SEM, n=12 tumors, * *P* < 0.01 vs. control.

*In-vivo* treatment of HCC1806 tumors with MIA-602, doxorubicin, or combination resulted in significant (P < 0.01) suppression of MDR1 and NANOG gene expression relative to controls. The expression of MDR1 in tumors treated with combination was more powerfully suppressed than with either compound alone (P < 0.001). Analysis of NANOG indicates that the combination treatment suppresses expression significantly (P < 0.001) more than either compound alone (figure 3a). Therapy of doxorubicin resistant MX-1 tumors with MIA-602 or combination led to significant (P < 0.05) reduction in MDR1 and NANOG gene expression relative to controls. The combination also significantly (P < 0.05) suppressed the expression of MDR1 and NANOG genes compared to doxorubicin alone, which did not have a significant effect (figure 3b).

### Lowering of efflux pump activity in HCC1806 and MX-1 human TNBC cells

**Figure 4:**
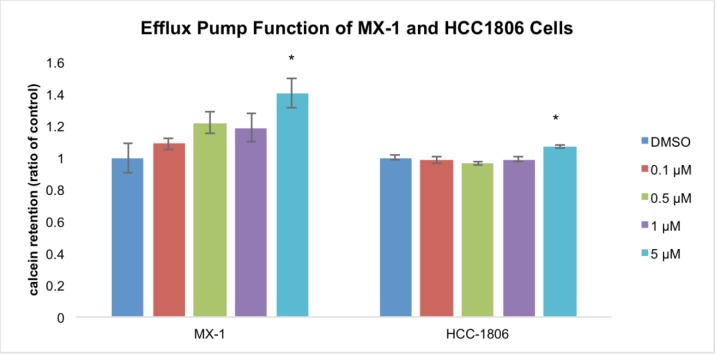
Efflux pump function assay TNBC cells treated with MIA-602 have significantly reduced efflux pump function compared to controls as evidenced by the retention of fluorescent dye. Vertical bars indicate ± SEM, n=9 samples, * *P* < 0.01 vs. control.

Efflux pump function was determined using a fluorescent dye (Calcein AM) retention assay. [40] The cells were treated with 5μM GHRH antagonist, MIA-602, for 4 hours and retention of Calcein was determined. HCC1086 cells and MX-1 cells, treated with 5 μM MIA-602, retained 7% and 40% more dye than controls, respectively. Both reductions in efflux pump function were significant compared to the respective controls (P < 0.01) (figure 4).

## DISCUSSION

Much information has now been accumulated concerning the role of GHRH, GHRH receptors, and receptor splice variants (SV) in carcinogenesis. The proliferation of various human cancers *in vitro* is suppressed by GHRH antagonists.[41] A report suggests that the dysregulation of GHRH expression or autocrine/ paracrine secretion contributes to the pathogenesis of breast and other cancers.[42] *In vivo* investiagtions have demonstrated the anti-tumor activity of GHRH antagonists against multiple cancer types. Studies on the effects of GHRH antagonists on prostate and lung cancers demonstrated their ability to modulate signaling pathways involved in cellular proliferation, survival, metastasis, and apoptosis.[43-45]

Increased expression of inflammatory cytokines correlates with higher tumor grade, greater metastatic potential, and higher incidence of resistance to treatment, all of which predict a poorer survival.[46] Among the roles of cytokines in breast cancer is their ability to regulate epithelial-mesenchymal transitions (EMT) and drug resistance.[38, 47, 48] In the course of EMT, expression of intercellular adhesion molecules, among other characteristics of an epithelial phenotype, is lost and cells acquire a stem-cell-like or “mesenchymal” phenotype. The resulting phenotype is highly motile and possesses stem-cell-like properties, a high degree of resistance to treatments, and an increased rate of drug efflux.[49-51]

Numerous studies have firmly established the regulatory role of inflammatory cytokines in cancer.[3, 37-39, 46, 47, 50, 52] Our group has recently reported the reduction in prostate size and suppression of inflammatory cytokines by GHRH antagonists in a rodent model of experimental benign prostatic hyperplasia (BPH).[53] We have also demonstrated similar effects of GHRH antagonists on *in vivo* cytokine gene expression by HCC1806 and MX-1 triple negative human breast cancer. Analysis of the genes expressed by tumors treated with MIA-602 indicates that it suppresses the expression of tumoral inflammatory cytokines.[36]

Studies on human cancer lines xenografted into nude mice have demonstrated anti-tumor activity of GHRH antagonists against multiple human cancer types. [6, 30, 54] Evaluations of GHRH antagonists in prostate and lung cancers demonstrated their ability to modulate signaling pathways involved in cellular proliferation, survival, metastasis, and apoptosis.[23, 43, 44, 55] Among the affected pathways is the PI3K-AKT, which regulates inflammatory cytokines through NF-κβ.[43, 44, 56] Activation of the NF-κβ pathway by inflammatory cytokines has also been shown to enhance drug resistance in breast cancer. Analyses indicate that treatment with GHRH antagonists suppresses the expression of pro-inflammatory cytokines in BPH.[13, 53]

Cell lines used in this study (HCC1806 and MX-1) were both susceptible to treatment with the GHRH antagonist, MIA-602. Tumors treated with MIA-602 showed a significant reduction in growth compared to controls. Combination of the GHRH antagonist with doxorubicin increased this inhibition of growth. Most importantly, MX-1 tumors, which are completely resistant to doxorubicin, were very responsive to the cytotoxic drug (doxorubicin) when it was administered in combination with MIA-602. Real-time RT-PCR analysis of the MX-1 tumors indicates that the expression of genes involved in drug resistance was reduced by treatment with MIA-602. The genes MDR1 and NANOG, both involved in the regulation and expression of efflux pump mechanisms, were significantly suppressed. Additionally, cells treated with MIA-602 showed a significant increases in Calcein dye retention, indicating that efflux pump function was reduced. These results illustrate the ability of GHRH antagonists to not only inhibit the growth of tumors, but also to enhance the effectiveness of other drugs by reducing resistance.

Several reports have detailed the effects of treatment with GHRH antagonists on the growth of breast cancers. [6, 20, 57, 58] The study presented herein illustrates the role of GHRH signaling in the regulation of drug resistance in TNBC. Our results also demonstrate the ability of a GHRH antagonist, MIA-602, to be a potential new treatment for drug resistant cancers. Our data suggest that changes in the expression of genes associated with drug resistance and cancer cell “stemness”, induced by treatment with MIA-602, lead to a significant reduction in the efflux capacity of drug resistant cells. This results in the accumulation of the cytotoxic drug in cells with disabled efflux mechanisms and reduces resistance. This study illustrates the potential benefits of using GHRH antagonists for the treatment of cancers that are highly resistant to cytotoxic agents such as doxorubicin.

## METHODS

### Drugs and Chemicals

The GHRH antagonistic analog, MIA-602, was synthesized in our laboratory as previously described. [7, 59] For daily subcutaneous injection, MIA-602 was dissolved in a 0.1% / 10% DMSO/propylene glycol solution. Doxorubicin was dissolved in a sterile solution of 5% mannitol for weekly intravenous injection. For *in-vitro* studies, both MIA-602 and doxorubicin were dissolved in DMSO to a concentration of 10mM and 2.5mM, respectively. The DMSO stock solutions were diluted with culture media keeping the final concentration of DMSO to less than 0.01%.

### Animals

Female nude mice (Harlan Laboratories) between 10 and 11 weeks of age (20g body weight) were housed in a climate-controlled environment with a 12-h light/ dark cycle and were fed standard laboratory diet with water *ad libitum*. Body weights were determined weekly. All animals remained healthy throughout the experiment. Animal care was in accordance with institutional guidelines and complied with National Institutes of Health policy.

### Cell Culture

Cultures of the human triple negative breast cancer cell line, HCC1806, were maintained in RPMI 1640 medium supplemented with 10% FBS. Flask cultures were kept in a humidified incubator in a 5% CO_2_ atmosphere at 37oC. Growth medium was replaced every 72 hours for two weeks. Cells were collected using 0.05% trypsin and incubating at 37oC for 3 minutes. Trypsin was inactivated with an equal volume of FBS containing medium and the cells were collected by centrifugation at 1000 × g for 10 minutes.

### Study Design

Donor animals were xenografted with 105 cells and tumors were allowed to grow for 4 weeks. The tumors were collected from euthanized animals and cut into approximately 5 mg fragments. Fragments were rinsed with sterile PBS and xenografted subcutaneously into both flanks of each animal. Tumors were allowed to grow to a mean volume of 75-100 mm^3^ prior to administration of the initial treatment. Animals were randomly assigned to one of four experimental groups. The control group remained untreated and the treated groups received daily subcutaneous injection of MIA-602 (5μg/day) and/or weekly intravenous (jugular) injections of doxorubicin (2.5nmol/kg/wk). Animals were treated for up to 5 weeks and tumor volume (mm3) = (length × width × height × 0.5236) and body weight were assessed weekly. Tumor inhibition (%) was calculated according to the following formula: [(final volume control - initial volume control) -(final volume treated - initial volume treated)]/(final volume control-initial volume control) × 100 [60]. All animals were euthanized by cervical dislocation upon study completion and tumors collected postmortem.

### RNA Isolation

Excised tumors were immediately cut into approximately 25 mg pieces and submerged in RNAlater (Ambion) stabilization solution. After an overnight incubation at 4oC, for thorough stabilization, samples were homogenized in lysis buffer and total RNA was isolated using the GE Illustra RNAspin Isolation Kit (GE Healthcare) according to the manufacturer's protocol. Contaminating DNA was eliminated with an on-column DNase treatment as part of the isolation procedure. Total RNA was quantified and assessed for purity using a Nanodrop spectrophotometer (Thermo Scientific).

### SYBR Green-based RT-PCR Primer Design

Gene expression was determined using qRT-PCR. All RNA targets were analyzed using custom designed oligonucleotide primers designed for use in SYBR green based qRT-PCR. The assays were painstakingly designed using extremely strict parameters in order to exclude non-human (mouse) templates and target regions of low energy secondary structures, maximizing both specificity and sensitivity. All assays were determined to produce a single product which was verified as the human target of interest by DNA sequencing.

Transcript specific primers were designed using the Beacon Designer software suite (Premiere Biosoft) with modified parameters. Primer searches were conducted to regions on mRNA sequences, obtained from the NCBI database, which were not homologous to the equivalent mRNA from mice (*Mus musculus*). The resulting human-specific sequences were screened for regions of stable secondary structures (ΔG < −3.0 Kcal/mol), which were excluded from our primer search. Primer searches were optimized for reverse transcription at 52oC and fast cycling polymerase chain reaction at 57oC. Primer hairpin energy was limited to ΔG = −3.0 Kcal/mol and dimer energies were limited to ΔG = −4.0 Kcal/mol. Dimers including the last 3 bases of the 3′ end of the primer were limited to ΔG = −2.0 Kcal/mol. Primers were designed to result in amplicons of 75-200 bp in length. Primer pairs that were less than 98% efficient were excluded. All primers used produce a single product of predictable and reproducible melting temperature (Tm). All primers were optimized and verified by sequencing the corresponding amplicons.

### Real-time Quantitative Reverse-Transcription Polymerase Chain Reaction (qRT-PCR)

Gene expression analysis was conducted using one-step qRT-PCR with SYBR green chemistry. This method conducts the reverse transcription reaction and PCR in a single tube format from 20ng total RNA template. The production of the PCR amplified gene product is monitored using the fluorescence resulting from the binding of SYBR green to the double stranded DNA amplicons. Reactions were conducted in a CFX96 Real-Time System using the One-Step SYBR Green qRT-PCR reaction kit (Bio-Rad). Reactions were conducted in triplicate and normalized to three internal standard genes using the δδCt method.[61]

### Efflux Pump Function Assay

Multidrug resistance of MX-1 and HCC-1806 cells was measured by calcein retention assay according to the manufacturer's instructions (Cayman Chemical) [62]. Briefly, cells were seeded onto 96-well plates to 20,000 cells/well density. Two days later, culture medium was replaced with Optimem (Life Technologies) containing 0.1 μM-5 μM MIA-602 for 4 hours. Calcein-AM and Hoechst were added at the end of the treatment for 10 minutes and the developed fluorescence was measured in assay buffer in a Victor3 plate reader (Hoechst at excitation and emission wavelengths of 355 nm and 465 nm, respectively and calcein at excitation and emission wavelengths of 485 nm and 535 nm, respectively). Values of calcein retention (efflux function) were normalized to Hoechst nuclear staining (viable cell density).

### Statistical Analysis

Prism 5 software (Graphpad Software, Inc.) was used for statistical evaluation of the data. Results are expressed as means ± SEM. One-way ANOVA followed by Bonferroni *t* test or a two-tailed Student's *t* test was used where appropriate, and significance was accepted at *P* < 0.05.
